# ACCEPTABILITY OF A CHATBOT FOR INFORMATION AND ADVICE ON DEMENTIA CAREGIVING

**DOI:** 10.1093/geroni/igae098.4107

**Published:** 2024-12-31

**Authors:** Sheung-Tak Cheng, Peter Ng, Tomoko Wakui

**Affiliations:** The Education University of Hong Kong, Tai Po, Hong Kong; Hong Kong Polytechnic University, Hong Kong, Hong Kong; Tokyo Metropolitan Institute for Geriatrics and Gerontology, Tokyo, Japan

## Abstract

Providing ongoing support to caregivers as their needs change in the long-term course of dementia is a serious challenge to any healthcare system. Conversational artificial intelligence (AI) operating 24/7 may help to tackle this problem. This presentation describes the development of a generative AI chatbot (called Chatbot-A) using the GPT-4o large language model, with a personality agent to constrain its behavior to providing advice on dementia caregiving based on a revised version of the Benefit-Finding Intervention manual (an intervention reported in JG:PS and other journals). The chatbot’s responses to 21 common questions were compared with those of another chatbot (called Chatbot-B) using a collection of authoritative sources (World Health Organization iSupport, By Us For Us Guides, and 185 webpages by Alzheimer’s Association, National Institute on Aging, and UK Alzheimer’s Society) as its knowledge base. Results showed highly context-sensitive and rather sophisticated answers by both chatbots, with Chatbot-A performing slightly better on mental health-related questions. Subsequently, 10 caregivers used Chatbot-A for two weeks and provided ratings and comments on its acceptability. They found Chatbot-A highly user-friendly, and its responses quite helpful and easy to understand. They were rather satisfied with it and would strongly recommend it to other caregivers. During the 2-week trial period, the majority used Chatbot-A more than once per day. Results supported conversational AI as a viable approach to improve support to caregivers. Due to space limitation, this poster will present the two chatbot’s answers to selected questions and the caregivers’ feedback on Chatbot-A in detail.

